# Body muscle mass versus fat mass: gender-specific associations with NAFLD and liver fibrosis

**DOI:** 10.1186/s12944-025-02694-4

**Published:** 2025-09-09

**Authors:** Konstantinos Volaklis, Dennis Freuer, Christa Meisinger, Jakob Linseisen

**Affiliations:** 1 Epidemiology, Medical Faculty, University of Augsburg, Stenglingstr. 2, Augsburg, 86156 Germany; 2 7FIT, Cardiac Rehabilitation Center, Augsburg, Germany

**Keywords:** Body muscle mass, Body fat mass, NAFLD, Liver steatosis, Gender, Liver fibrosis

## Abstract

**Background:**

This study aimed to investigate the gender-specific associations of skeletal muscle mass and fat mass with non-alcoholic fatty liver disease (NAFLD) and NAFLD-related liver fibrosis in two population-based studies.

**Methods:**

Analyses were based on data from the MEGA (*n* = 238) and the MEIA study (*n* = 594) conducted between 2018 and 2023 in Augsburg, Germany. Bioelectrical impedance analysis was used to evaluate relative skeletal muscle mass (rSM) and SM index (SMI) as well as relative fat mass (rFM) and FM index (FMI); furthermore, the fat-to-muscle ratio was built. The fatty liver index (FLI) was calculated to identify NAFLD. To estimate the degree of liver fibrosis, liver stiffness was assessed in the MEGA study using ultrasound elastography. Multivariable gamma regression models with log-link were used to analyze the respective associations.

**Results:**

After adjusting for confounders, FMI and rFM (*p* < 0.001) as well as SMI (*p* < 0.001) were significantly positive associated with FLI. The associations were non-linear, and effect modification by gender (p_gender−interaction_ < 0.001) existed in all models except for SMI, while age modified the rSM and SMI results. The effect estimates for FMI and rFMI were higher in men than in women but approached each other at the high FMI range. Increasing rSM was inversely associated with FMI. The fat-to-muscle ratio was positively associated with FLI in men and women. However, no associations were found between the exposure variables and liver fibrosis.

**Conclusions:**

This population-based study demonstrated gender-specific positive associations between fat mass parameters and FLI, and an inverse association with rSM. Furthermore, for rSM effect modification by age was observed. Neither in men nor in women associations between body composition and liver fibrosis could be identified.

**Supplementary Information:**

The online version contains supplementary material available at 10.1186/s12944-025-02694-4.

## Introduction

In the past decades, the prevalence of non-alcoholic fatty liver disease (NAFLD), also called metabolic dysfunction-associated fatty liver disease (MAFLD), strongly increased worldwide mainly as a result of the obesity and type-2 diabetes epidemic [1]. NAFLD is characterized by excessive fat accumulation in hepatocytes (hepatic steatosis) which may develop into steatohepatitis, liver fibrosis, and eventually liver cirrhosis [2]. The global prevalence of NAFLD in adults is roughly estimated to be one-third, varying between 21 and 44% according to different classifications, subtypes, and applied methodologies [3, 4].

Due to a strong association between NAFLD, metabolic derangements, cardiovascular disease, and premature mortality [5, 6], the early detection and treatment of NAFLD is of great importance for public health and the healthcare systems. Despite the progress of invasive and non-invasive diagnostic tools, major efforts for specific and effective treatment of NAFLD are warranted [7]. So far, reduction of excess body weight is the major therapeutic target [8].

Recently, Soler et al. [9] evaluated the association of different scales of overweight and obesity with several fatty liver indices. The authors concluded that body fat and visceral fat (measured by bioelectric impedance analysis) were even stronger associated with NAFLD than body mass index (BMI) and waist circumference. In contrast to the expectations, there was no clear difference between visceral fat mass and total fat mass in predicting NAFLD. This was confirmed by other studies not demonstrating a better prediction of NAFLD by visceral fat measures as compared to relative fat mass [10].

Body composition differs in men and women. The proportion of body fat ranges between 6-15% and 9–23% for normal-weight men and women, respectively [11]. Gender-related differences in the pathophysiology of NAFLD were described but the reasons for this are not entirely understood. Several factors (e.g. genetic, hormonal, socio-cultural) that differ by gender may impact hepatic lipid accumulation [12]. In women, menopausal status seems to have a strong impact on NAFLD development which occurs most often among postmenopausal women [13]. So far, inconsistency exists regarding the gender effects on advanced stages of NAFLD such as steatohepatitis and liver fibrosis [14–16]. A cross-sectional study on the evaluation of the relationship between total fat and body fat distribution and NAFLD based on NHANES data showed that an android fat deposition pattern was associated with liver fibrosis only in women [15]. Myake et al. found that fat mass indices were stronger associated with Liver fibrosis diagnosed by Liver biopsy than muscle mass in a sample of 157 patients with NAFLD [17]. However, in that study no gender-specific analyses were conducted.

Reduced body muscle mass is an independent factor adversely affecting the risk of NAFLD development and progression [18, 19]. According to the results of a recent systematic review including 14 randomized controlled trials (RCTs), exercise training is likely to achieve clinically meaningful treatment response in MRI-measured liver fat, independent of weight loss [20]. These results suggest independent benefits of physical training, such as changes in body composition with loss of adipose tissue and increase in lean muscle mass. Further studies are needed to explore the interplay between fat and muscle mass for excessive liver fat accumulation and the progression to liver fibrosis in the general population.

Thus, the present study focused on both, fat mass and muscle mass using bioelectric impedance analysis (BIA) assessed measures including the fat-to-muscle ratio, and their relationship to NAFLD in men and women from the general population. Furthermore, the gender-specific associations between the different exposures and NAFLD-related fibrosis were investigated.

## Methods

### Study design

This analysis was based on data from the MEGA study (German acronym for metabolic health study), which was conducted between 2018 and 2021 and the MEIA study (German acronym for metabolism, diet, and immune system), which was carried out between 2021 and 2023 in Augsburg, Germany.

In the MEGA study, participants from the general population in the Augsburg study region (city of Augsburg, county of Augsburg, and county of Aichach-Friedberg) aged 25 to 65 years were recruited and examined up to four times within a period of 9 months. The main objectives of the study were to examine immunological particularities that are associated with anthropometric measures, diet, and Lifestyle. To analyse the impact of obesity, oversampling of obese subjects was performed. Study participants were recruited in various ways, e.g. through posters and flyers, announcements in newspapers and social media, and examined in the study center of the Chair of Epidemiology. The examinations included, amongst others, a personal interview, completion of self-administered questionnaires, collection of a fasting venous blood sample, anthropometric measurements and BIA, and Liver elastography. A total of 238 MEGA participants were examined at recruitment.

In the MEIA study, 594 randomly selected participants from the Augsburg study region, i.e. the same region as selected for the MEGA study, aged between 18 and 75 were examined at the study center of the Chair of Epidemiology at the University Hospital Augsburg. The addresses were provided by civil registries of involved communities. Overall, 19% of the invited subjects participated. In the MEIA study, information was collected in a face-to-face interview and with the help of questionnaires (self-administered) (e.g. on sociodemographic information, smoking status, alcohol consumption). Fasting blood samples were collected and the participants underwent a comprehensive examination program with anthropometric examinations, blood pressure measurements, etc.

Both studies used similar instruments, questionnaires, and physical measurements to collect data. Therefore, data on anthropometric measurements, blood parameters, and lifestyle factors were pooled. This resulted in an enrichment of obese subjects in the pooled data set. As participants in both studies were recruited from the same study population and examined in the study center with the same methods, instruments, and trained staff, the pooled analysis is justified. Inclusion criteria were the same in both studies, i.e. meeting the given age range, main residence in the study area, and ability to provide informed consent; we excluded institutionalized subjects.

The Ethics Committee of the Ludwig-Maximilians-Universität München approved both studies. The investigations were carried out in accordance with the Declaration of Helsinki, including written informed consent of all participants. Both studies were registered at “Deutsches Register Klinischer Studien” (DRKS) with the project number DRKS00015784 (MEGA study) and the project number DRKS00028738 (MEIA study).

### Data collection

Patient information was obtained during a face-to-face interview and by completing questionnaires. Besides sex and age, information on smoking was assessed and transformed to never/ex/current smoking and pack-years of smoking (quantifying the amount and duration of smoking). For information on (risky) alcohol consumption, the AUDIT-C questionnaire data was used to calculate the AUDIT-C score and ethanol intake (g/d). Physical activity was represented by the Metabolic Equivalent of Task (MET) by calculating the MET-minutes/week. This enabled the physical activity of the participants to be compared. The information on the highest education level and occupational training were combined to establish the ISCED classification [21]. For grouping into low, middle, and high SES groups, ISCED levels 1 and 2 (low), 3 and 4 (middle), and 5 and 6 (high) were combined.

### Exposures

Next to the measurement of body weight and height, BIA measurements (resistance, reactance, and phase angle) were obtained using the Seca mBCA 515 device; by means of the Seca 115 software estimates of total fat mass (FM, in kg) skeletal muscle mass (SM, in kg) were obtained. Relative body fat mass (rFM, in % of body weight), relative skeletal muscle mass (rSM, in % of body weight), as well as fat mass index (FMI; FM/squared body height; kg/m²) and skeletal muscle mass index (SMMI; SMM/squared body height; kg/m²) were calculated [22]. In addition, the fat-to-muscle ratio (FMR) was estimated. Finally, in sensitivity analyses waist circumference (WC) was used to assess the impact of visceral fat content.

### Outcomes

The Fatty Liver-Index (FLI) was calculated according to Bedogni et al. [23] as follows:

$$\:FLI=\frac{100\cdot\:exp\left(0.953\:*\:log\right(TG)\:+\:0.139\:*\:BMI\:+\:0.718\:*\:log(GGT)\:+\:0.053\:*\:waist\:-\:15.745)}{1+exp\left(0.953\:*\:log\right(TG)\:+\:0.139\:*\:BMI\:+\:0.718\:*\:log(GGT)\:+\:0.053\:*\:waist\:-\:15.745)}$$

where BMI denotes body mass index (kg/m²), TG denotes triglycerides, and GGT denotes γ-glutamyl transferase. The FLI (given in %) varies between 0 and 100.

Liver elastography was carried out only in participants of the MEGA study. A change in the elasticity of the liver parenchyma can be assumed when fat is stored and when connective tissue forms. This altered stiffness is measured quantitatively using elastography, which makes it possible to assess liver stiffness in addition to B-imaging. There are various elastographic procedures [24]. In the MEGA study, elastography was carried out with the ultrasound device EPIQ 7G from Philips, which enables the measurement using acoustic radiation force impulse (ARFI) technology. With the ARFI technique, short acoustic pulses are emitted into the examined tissue inducing tiny tissue displacements. These displacements lead to the propagation of transverse waves away from the excitation region. The speed at which these transverse waves propagate is in turn detected and measured using ultrasound. The stiffer the tissue, the faster the propagation speed. The elastography was carried out on a fasting participant in the area of Liver segment 8. Ten measurements were carried out and the mean value of these measurements was calculated [25]. Liver elastography has been validated against liver biopsy, which is the gold standard method for fibrosis diagnosis [26]. In the present study, only participants of the MEGA study were included in the analysis regarding the outcome liver pressure.

### Statistical analysis

In the descriptive analyses, continuous variables were presented as median and interquartile range (IQR), and differences by gender or FLI categories were tested using the non-parametric two-sided Wilcoxon Rank Sum test and the Kruskal-Wallis Rank Sum test, respectively. Categorical variables were expressed as absolute and relative frequencies and tested for group differences using the $\:{\chi\:}^{2}$ test. Multivariable gamma regression models with log-link were used for the analysis of associations. This regression method provided very similar estimates to the log-OLS model but was the preferred model because of the better interpretation of coefficients and 95% confidence intervals. Restricted cubic splines were used to ensure the linearity assumption between continuous covariables and the log-transformed outcomes. The number of knots for each continuous variable-outcome association was determined based on the AIC. Model-specific outliers were quantified using Cook’s distance and, if necessary, omitted from the respective analysis.

Potential confounders were selected using DAGs (directed acyclic graphs; see Supplementary Figure S1). Age, pack-years of smoking, MET-minutes/week, and the AUDIT-C score were used as continuous covariables. Gender (female, male), education (classified according to ISCED in low, middle, and high), and smoking status (current, previous, never) were used as categorical variables. Since the FLI models were conducted in the combined data set from the MEGA and the MEIA study, the respective study was used as an additional confounder after testing for interaction effects with the respective exposure. Sensitivity analyses were performed by applying all models exclusively to data of the MEIA study sample to test robustness of the results.

Due to differences in body composition and notable differences especially in FLI distributions, further interaction effects between exposures and gender as well as age (continuous variable) were tested. Since all missing values could be assumed as completely missing at random, a complete case analysis was performed. *P* values from regression analyses were Bonferroni-adjusted to account for multiple testing. All analyses were conducted using the open-source statistical software R (version: 4.3.2).

## Results

The baseline characteristics of the 796 study participants, stratified by gender, are shown in Table 1. The median age of the combined studies was 49 years and 40% were men. Compared to women, men had a higher median BMI and waist circumference but also a higher skeletal muscle mass and a lower relative fat mass. For the sake of completeness, the study-specific characteristics stratified by gender are presented in the Supplementary Tables S1 and S2.

**Table 1:** Characteristics of the study participants of the total analytic sample (MEIA and MEGA pooled) and stratified by gender. Continuous variables are given as median and interquartile range and categorical variables are presented as absolute and relative frequencies

Characteristics	Total	Females	Males	*p* value*
	*n* = 796	*n* = 479	*n* = 317	
Age (years)	49 (35; 59)	49 (35; 57)	50 (35; 61)	0.206
BMI (kg/m²)	25.5 (22.64; 30.26)	24.28 (21.26; 29.12)	27.005 (24.548; 30.87)	< 0.001
Waist circumference (cm)	87 (75.625; 100)	80 (71; 92.75)	96 (86; 106)	< 0.001
Fatty liver index	24.348 (6.975; 65.948)	12.419 (4.672; 46.106)	53.01 (20.185; 80.936)	< 0.001
Liver pressure (kPa)	5.245 (4.908; 5.78)	5.24 (4.898; 5.69)	5.345 (4.91; 6.122)	0.24
Fat mass (kg)	22.523 (16.61; 31.874)	22.924 (16.822; 32.407)	22.022 (16.479; 30.837)	0.184
Relative fat mass (%)	31.165 (25.47; 37.85)	35.24 (28.41; 41.47)	26.39 (21.175; 32.115)	< 0.001
Fat mass index (kg/m²)	7.69 (5.71; 10.803)	8.33 (6.11; 11.79)	7 (5.185; 9.675)	< 0.001
Skeletal muscle mass (kg)	23.645 (19.578; 30.432)	20.29 (18.25; 22.53)	31.4 (28.18; 34.7)	< 0.001
Relative skeletal muscle mass (%)	32.132 (28.346; 35.331)	29.443 (26.52; 32.317)	35.878 (33.337; 38.279)	< 0.001
Skeletal muscle mass index (kg/m²)	8.191 (7.115; 9.653)	7.33 (6.707; 8.092)	9.728 (9.041; 10.614)	< 0.001
Fat-to-muscle ratio	0.978 (0.723; 1.315)	1.199 (0.879; 1.564)	0.739 (0.556; 0.963)	< 0.001
Triglycerides (mg/dl)	88 (67; 126)	80 (62; 111)	105 (74; 145)	< 0.001
Gamma-glutamyl transferase (U/l)	19 (13; 29)	15 (12; 21)	25 (19; 38)	< 0.001
AUDIT-C score	3 (1; 4)	2 (1; 4)	4 (2; 5)	< 0.001
Smoking: pack-years	0 (0; 6.213)	0 (0; 4.8)	0.105 (0; 9)	0.007
Physical activity (MET-minutes/week)	4182 (2193; 6091)	3872 (1827; 5690)	4700 (3042; 6333)	0.005
Obesity				0.062**
BMI ≥ 30 kg/m²) (n, %)	204 (26)	111 (23)	93 (29)	
BMI < 30 kg/m²) (n, %)	592 (74)	368 (77)	224 (71)	
Diabetes				0.073**
Yes, (n, %)	17 (2)	7 (1)	10 (3)	
No, (n, %)	400 (50)	262 (55)	138 (44)	
Education				0.193**
High, (n, %)	298 (37)	171 (36)	127 (40)	
Middle, (n, %)	28 (4)	14 (3)	14 (44)	
Low, (n, %)	470 (59)	294 (61)	176 (56)	

*AUDIT-C* Alcohol use disorders identification test consumption, *BMI* Body mass index, *Pack-years* Packs per day x number of years smoked

*Wilcoxon Rank Sum test, if not otherwise specified

**Pearson’s Chi-squared test

Table 2 shows the characteristics of the subjects according to three FLI-based categories: no fatty liver (FLI < 30), existing fatty liver (FLI > 60), and a group in-between (possibly fatty liver). Subjects with fatty liver were more often male, showed a higher BMI and waist circumference and had the highest fat mass (in kg and in %) and FMI. However, skeletal muscle mass (in kg) and SMI was highest in this group, though rSM was significantly lower as compared to the two other liver fat groups. In addition, subjects with fatty liver revealed higher median systolic blood pressure, higher serum triglycerides, and higher liver enzyme values as found for participants with lower FLI values.

**Table 2:** Characteristics of the participants of the total analytic sample (MEIA and MEGA pooled) by categories of fatty liver index (FLI). Continuous variables are given as median and interquartile range and categorical variables are presented as absolute and relative frequencies

	Fatty Liver Index			
	No	Possibly	Yes	
Characteristics	(FLI < 30)	(FLI = 30–60)	(FLI > 60)	
	*n* = 411	*n* = 125	*n* = 206	*p* value*
Age (years)	44 (32; 55)	54 (42; 62)	50 (39; 60)	< 0.001
BMI (kg/m²)	22.94 (20.89; 24.62)	27.31 (26.02; 28.83)	32.92 (30.43; 37.26)	< 0.001
Waist circumference (cm)	76.5 (70.5; 82.0)	95 (90; 100)	107 (101; 115)	< 0.001
Fat mass (kg)	17.67 (14.35; 22.14)	26.45 (21.60; 31.78)	38.03 (29.66; 48.40)	< 0.001
Relative fat mass (%)	28.21 (22.97; 33.93)	31.57 (25.79; 40.79)	36.52 (31.14; 46.81)	< 0.001
Fat mass index	6.19 (5; 7.99)	8.55 (6.77; 11.57)	12.27 (9.57; 16.48)	< 0.001
Skeletal muscle mass (kg)	20.61 (18.33; 24.38)	26.5 (21.35; 30.54)	31.23 (26.41; 34.86)	< 0.001
Relative skeletal muscle mass (%)	32.45 (29.61; 36.14)	33.41 (26.80; 35.97)	31.20 (25.53; 33.90)	< 0.001
Skeletal muscle mass index	7.33 (6.70; 8.20)	8.80 (7.89; 9.55)	10.20 (9.25; 10.98)	< 0.001
Fat-to-muscle ratio	0.88 (0.64; 1.14)	0.95 (0.73; 1.51)	1.16 (0.92; 1.83)	< 0.001
Liver pressure (kPa)	5.24 (4.92; 5.70)	5.28 (5.01; 5.98)	5.20 (4.86; 5.83)	0.504
Triglycerides (mg/dl)	72.0 (56; 88)	105.0 (79.0; 130.0)	137.0 (99.5; 175.5)	< 0.001
Gamma-glutamyl transferase (U/l)	15.0 (11.5; 20.0)	22.0 (17.0; 27.0)	31.0 (22; 48)	< 0.001
AUDIT-C score	3 (2; 4)	3 (1; 4.5)	3 (1; 5)	0.453
Smoking (Pack-years)	0 (0; 3.60)	0.28 (0; 7.98)	0.1 (0; 10.76)	< 0.001
Smoking				0.042 **
Current, (n, %)	60 (14)	17 (13)	37 (17)	
Never, (n, %)	239 (57)	58 (46)	105 (48)	
Previous, (n, %)	119 (29)	52 (41)	78 (36)	
Physical activity (MET-minutes/week)	4564 (2748; 6164)	3839 (1982; 6096)	3625 (1440; 5543)	0.015
Obesity				< 0.001**
BMI ≥ 30 kg/m²) (n, %)	4 (0.97)	23 (18.4)	177 (85.9)	
BMI < 30 kg/m²) (n, %)	407 (99.0)	102 (81.6)	29 (14.1)	
Diabetes				< 0.001**
Yes, (n, %)	2 (0.49)	2 (1.60)	13 (6.31)	
No, (n, %)	231 (56.2)	57 (45.6)	109 (52.9)	
Education				0.002 **
High, (n, %)	180 (43.8)	48 (38.4)	59 (28.6)	
Middle, (n, %)	223 (54.3)	72 (57.6)	152 (67.0)	
Low, (n, %)	8 (1.9)	5 (4.0)	11 (5.3)	
Gender				< 0.001**
Females, (n, %)	307 (74.7)	56 (44.8)	80 (38.8)	
Males, (n, %)	104 (25.3)	69 (55.2)	126 (61.2)	

*AUDIT-C* Alcohol use disorders identification test consumption, *BMI* Body mass index, *Pack-years* Packs per day x number of years smoked)

*Kruskal-Wallis Rank Sum test, if not otherwise specified

**Pearson’s Chi-squared test

The estimates obtained from the gamma regression models can be interpreted as the percentage change in an outcome (compared to the 1 as reference) with an increase in the respective exposure by one unit.

**Fig. 1:**
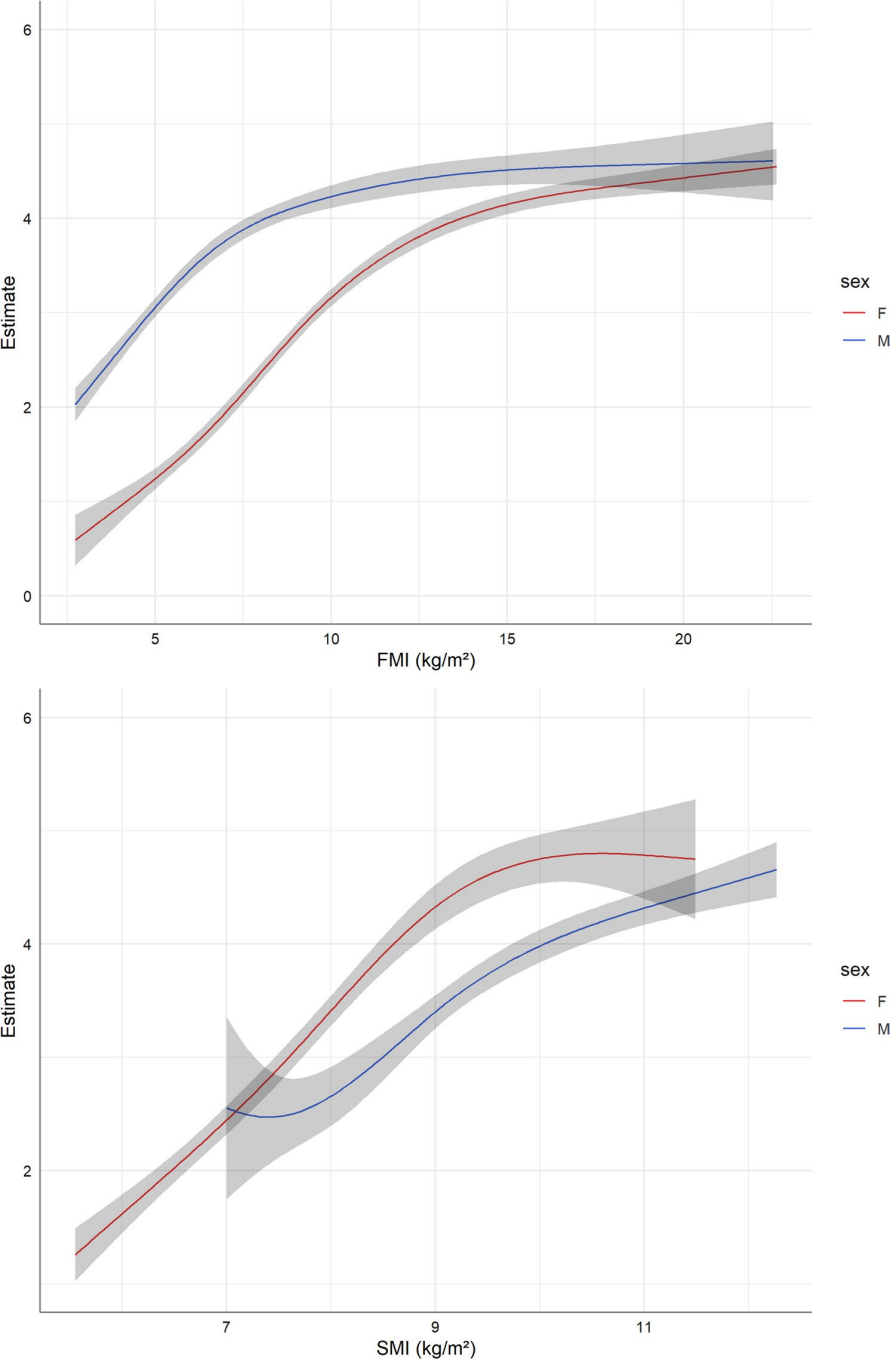
Gender-specific non-linear associations between fat mass index (FMI) as well as skeletal muscle mass index (SMI) and fatty liver index (FLI) estimated by restricted cubic splines within gamma regression models. The slopes of the estimates at each point represent the percentage change when increasing FMI or SMI by one unit

After adjusting for several confounders, FMI (*p* < 0.0001) was significantly related to FLI (Table 3). The association was non-linear and differed significantly between men and women (p_gender−interaction_ < 0.0001). The positive associations curves between FMI and FLI were strongest in men with small FMI values (58% change increase per 1-point increase in FMI between 1 and 3) and in women with medium values (53% change increase per 1-point increase for FMI of 7 vs. 8) (Fig. 1).

**Table 3:** Estimates and 95% confidence intervals from multivariable gamma regression models for the relationships between body composition variables (FMI, SMI, rFM, rSM, FMR) with fatty liver index (FLI) and liver fibrosis (liver pressure). The estimates on the exponential scale can be interpreted as percentage change with the 1 as reference. P values for the estimated effects were Bonferroni adjusted. (Results of the non-linear associations are graphically shown in Figs. 1, 2 and 3)

Exposure	Outcome	Estimate (95% CI)	*P*	*P* gender-interaction	*P* age-interaction
FMI	FLI	non-linear (4 knots)	< 0.0001	< 0.0001	0.6666
rFM	FLI	non-linear (4 knots)	< 0.0001	< 0.0001	0.5422
SMI	FLI	non-linear (3 knots)	< 0.0001	0.1154	< 0.0001
rSM	FLI	non-linear (3 knots)	< 0.0001	0.0009	0.0070
FMR	FLI	non-linear (3 knots)	< 0.0001	0.0001	0.0996
FMI	Liver Pressure	1.001 (0.995; 1.008)	1	0.3375	0.9571
rFM	Liver Pressure	1.001 (0.998; 1.004)	1	0.2431	0.9843
SMI	Liver Pressure	0.998 (0.974; 1.022)	1	0.3112	0.4544
rSM	Liver Pressure	0.998 (0.974; 1.023)	1	0.3352	0.7082
FMR	Liver Pressure	1.026 (0.948; 1.109)	1	0.3734	0.7760

*FMI* Fat mass index (kg/m²), *rFM* relative fat mass (% body weight), *SMI* Skeletal muscle mass index (kg/m²), *rSM* relative skeletal muscle mass (% body weight), FMR fat-to-muscle ratio

**Fig. 2:**
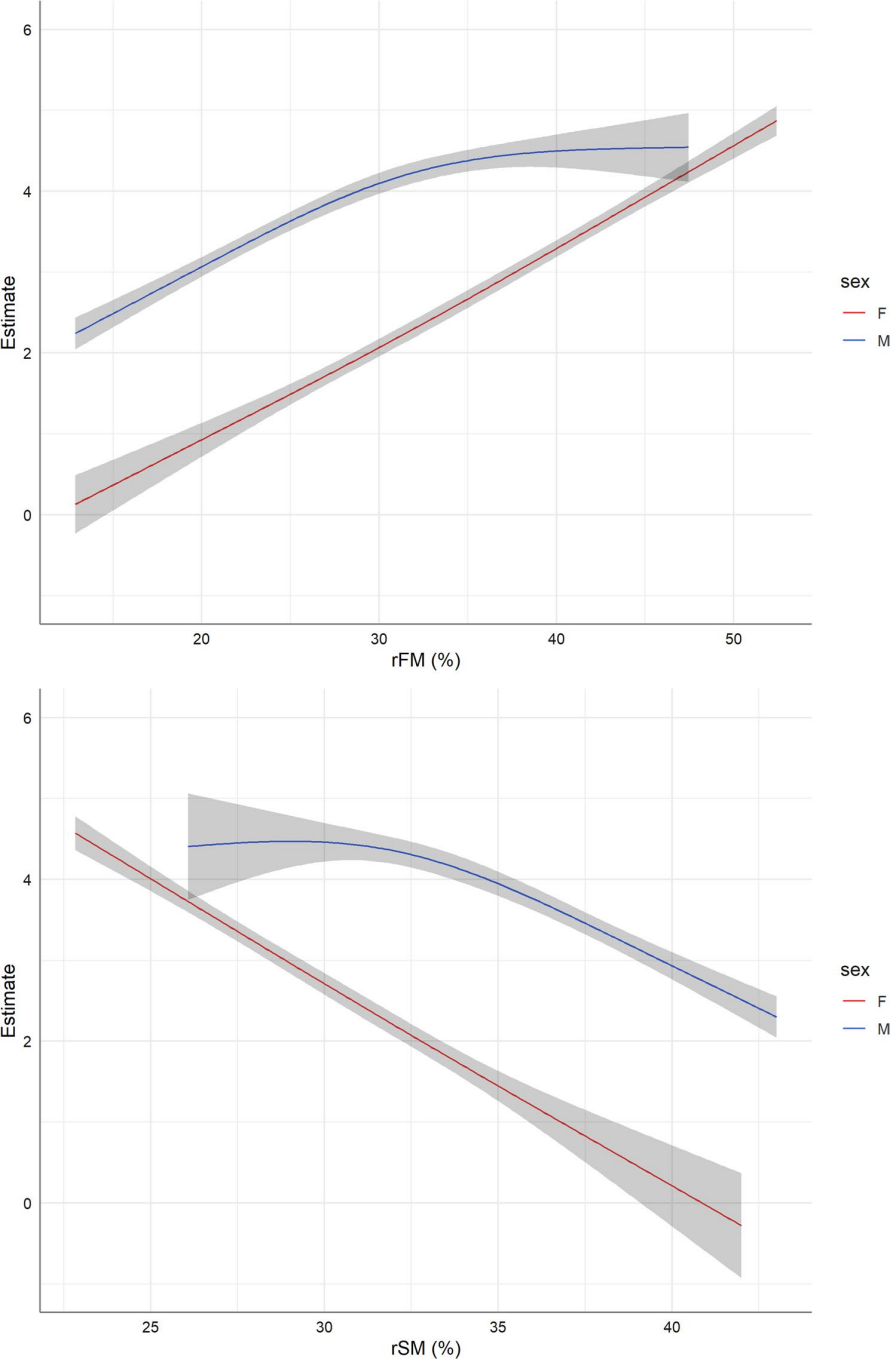
Gender-specific non-linear associations between relative fat mass (rFM, % body weight) as well as relative skeletal muscle mass (rSM, % body weight) and fatty liver index (FLI) estimated by restricted cubic splines within gamma regression models. The slopes of the estimates at each point represent the percentage change when increasing rFM or rSM by one unit

**Fig. 3:**
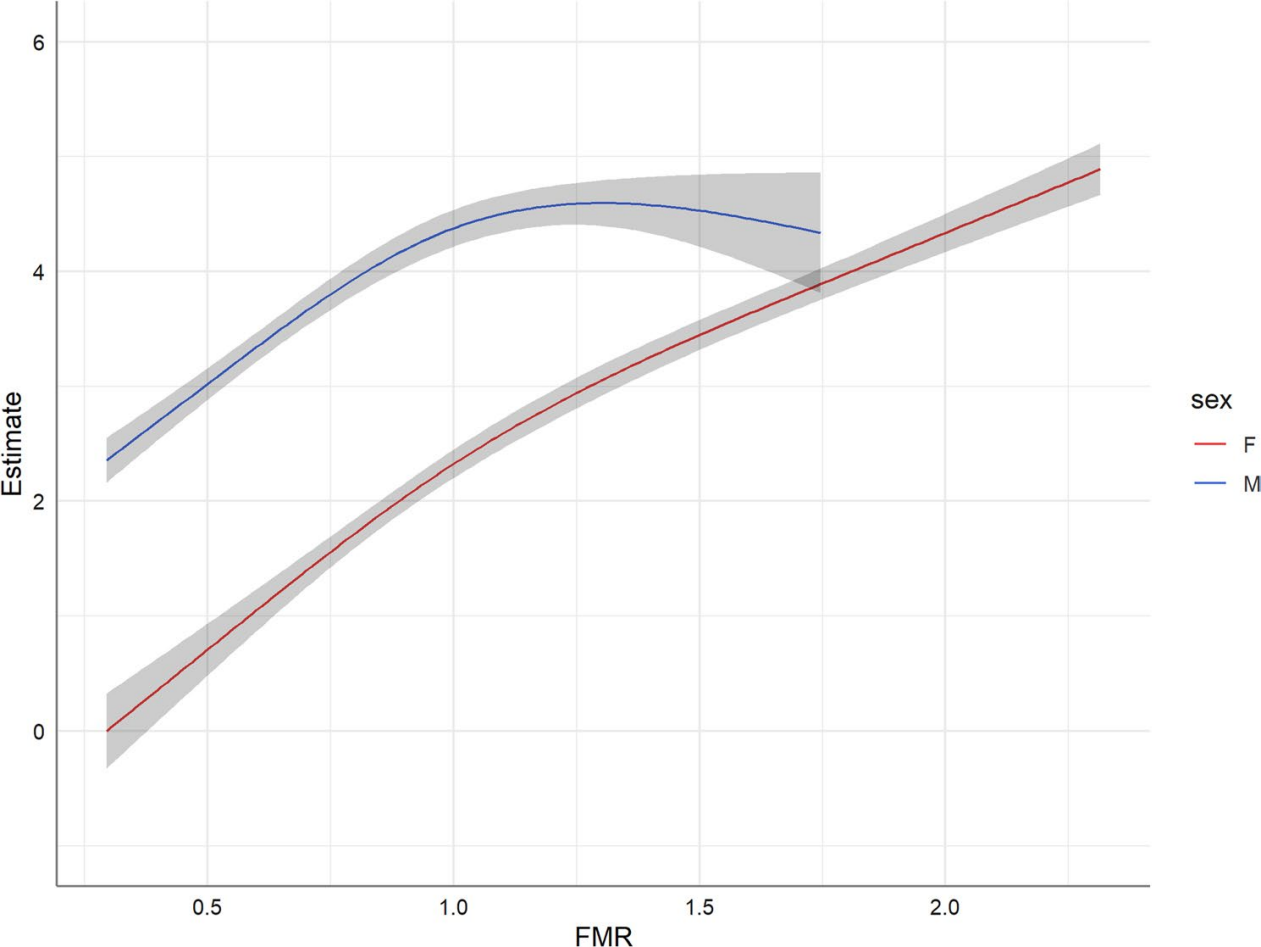
Gender-specific non-linear associations between fat-to-muscle ratio (FMR) and fatty liver index (FLI) estimated by restricted cubic splines within a gamma regression model. The slopes of the estimates at each point represent the percentage change when increasing FMR by one unit

SMI was also positively related to FLI in both genders but appeared to be modified by age (Table 3; Fig. 1). However, stratified analyses with a cut-off value of 50, also as a proxy for pre- versus peri- and post-menopausal status in women, showed similar associations for both age groups (Supplementary Figure S2).

We also examined the non-linear associations of rFM, rSM and FMR with FLI, adjusted for several confounders (Figs. 2 and 3). There was a positive association between both rFM and FMR with FLI while rSM was inversely related to FLI. For all exposures, the associations with FLI were non-linear and differed significantly between men and women (p_gender−interaction_ < 0.0001, Table 3). Despite a significant interaction with age (p_age−interaction_ = 0.007, Table 3), the gender-specific rSM-FLI associations were similar for individuals under and over 50 (Supplementary Figure S3).

The associations with FLI were fully confirmed by the sensitivity analyses considering participants from the MEIA study only (Supplementary Table S3, Supplementary Figure S4). Results from additional analyses for the association between WC and FLI is presented in Supplementary Table S4 and Supplementary Figure S5. In particular, there was no evidence of age- or gender-specific differences in the non-linear association between WC and FLI.

No associations were observed between any assessed exposure and liver pressure (Table 3, Supplementary Table S4).

## Discussion

According to literature, obesity and reduced muscle mass are two important pathogenic factors that contribute to an increased risk of NAFLD and NAFLD-related fibrosis [7, 8]. However, inconsistency exists about the impact of gender on these associations, in particular regarding liver fibrosis [15, 17]. In the present study, all investigated obesity and muscle mass measures were associated with NAFLD defined by the FLI. The associations were stronger for men than for women (except for SMI). The rSM mass was inversely related to FLI and the association was modified by both gender and age. However, no relationship between the investigated measures and liver fibrosis were found in men and women.

The stronger association between FMI and FLI observed in men seems paradoxical given that women have higher percentages of body fat compared to men [27]. However, recent studies suggested that body fat distribution (and not general obesity) is the critical factor since visceral fat was stronger associated with NAFLD than subcutaneous adipose tissue [28, 29]. It is also known that in men fat accumulates more in the abdomen area (android obesity) and this type of fat distribution is often accompanied with ectopic fat deposition (in skeletal muscles and liver) and is associated with a higher risk for insulin resistance and cardiometabolic diseases [30, 31].

In addition, it is well accepted that hormonal differences affecting liver metabolism and serum lipoprotein levels are responsible for the reduced risk of hepatic steatosis of women and the higher levels of endogenous estrogens has been proposed as the main mechanism for this [12]. Indeed, higher fat oxidation rate, faster plasma clearance of fatty acids, a lower postprandial lipid accumulation as well as a better regulation of de novo lipogenesis in females have been reported [32, 33]. Thus, the stronger association between FMI and FLI as observed in men follows this line of arguments. Τaken together, women appear to have some protection against NAFLD as this was additionally shown in the associations between rFM and FMR with FLI.

We further observed a strongly negative, nearly linear association between rSM in women while in men this relationship was non-linear meaning that the protective role of skeletal muscles regarding NAFLD risk may occur in men at higher levels of muscle mass (see Fig. 2). One possible explanation for this are the different androgen levels in men and women given that testosterone plays an important role in protein metabolism and skeletal muscle development in men [12]. Data from cross-sectional studies suggested that low testosterone levels are strongly associated with NAFLD independent of insulin resistance or levels of body fat and that normal androgen levels prevent hepatic fat accumulation and steatosis (hypogonadism hypothesis) [34, 35]. Furthermore, in this study men were overweight having a mean BMI of 27.0 kg/m² (compared to 24.3 kg/m² in women) and given that obesity is an important risk factor for secondary hypogonadism in men [36] the different observed association regarding skeletal muscle mass and NAFLD between genders are, at least partly, explainable.

The fat-to-muscle ratio increases with increasing body fat content or decreasing muscle mass. In this study, including participants from the general population, strongly reduced body mass as a consequence of illness may rarely be present, thus increasing fat mass seems the driver here (Fig. 3). Indeed, the association curve was similar to the rFM curve in men and women (Fig. 2).

In our study we observed no association between FMI and SMI and viscoelastic parameters of hepatic fibrosis (in both genders) and this is in line with some [14, 37] but not all prior studies [15, 38, 39]. In the study of Ciardullo et al. [15], a significant association between android to gynoid ratio (an index of body fat distribution) and liver fibrosis was observed only in females while in the study of Li et al. [14] lower skeletal muscle mass combined with abdominal obesity was strongly associated with biopsy-proven NAFLD only in males. A recent meta-analysis showed that although males compared to females are at increased risk of NAFLD, once NAFLD has occurred, females are at higher risk of progression toward advanced liver fibrosis [40]. Other studies also reported the protective effects of estrogen on fibrinogenesis [16]. Still, there is no consensus in the literature and additional studies examining the gender-specific associations of muscle mass and body fat in advanced stages of NAFLD are needed.

### Limitations

The BMI (body weight/height^2, kg/m²) is included in the estimation of the fatty liver index, and FMI and SMI are calculated as two major parts of the BMI, and both increase with increasing BMI. This explains why increasing SMI is associated with increasing FLI. Thus, expression of fat and muscle mass in % of body weight is the preferred estimate when using FLI as an outcome variable. The FLI developed by Bedogni’s group in 2006 [23], has been validated as a reliable biomarker for identifying NAFLD [41]. But the diagnosis of NAFLD based on FLI and not by ultrasound or biopsy is a shortcoming, because the higher waist circumferences and BMI levels in men may influence the FLI values and thus partly explain the observed gender differences found in the present study. However, no gender-differences were found when analyzing the association between waist circumference and FLI in this study. Due to the cross-sectional character of our study, we were not able to determine causal associations; prospective cohort studies are needed to validate our results. Although we adjusted for several measured confounders using the DAG approach, bias regarding unmeasured factors (such as menopausal status or sex hormone levels) could not be completely ruled out. Furthermore, the analyses in this study were based on measurements obtained using BIA to determine body fat compartments. Although this approach is better than using BMI, computer tomography (as the gold standard) would lead to more precise results. Also, since liver elastography was only used in the MEGA study and not in the MEIA study, the results of the subsequent subgroup analyses may suffer from low statistical power. Finally, although transient elastography is a highly repeatable method, is not as specific as liver biopsy for the accurate assessment of liver fibrosis.

In conclusion, in this study markers of body fat content (rFM, FMI) and the fat-to-muscle ratio were positively related to NAFLD with the associations being stronger for men than for women after adjusting for several confounding factors. Furthermore, relative skeletal muscle mass was inversely related to excessive liver fat content in both genders. However, neither in men nor in women associations were found between measures of body fat or muscle mass and liver fibrosis. Prospective cohort studies are required to quantify the effects of combinations of high/low muscle mass and a high fat mass in relation to fatty liver development in men and women, and in different age groups.

## Supplementary Information

Supplementary Material 1.

Supplementary Material 2.

## Data Availability

The datasets generated during and/or analyzed during the current study are available from the corresponding author on reasonable request.
