# Supplementary material for: Body muscle mass versus fat mass: gender-specific associations with NAFLD and liver fibrosis
Source: Lipids Health Dis. 2025 Sep 9;24:278. doi: 10.1186/s12944-025-02694-4 (PMC12418690; doi:10.1186/s12944-025-02694-4)
Supplement: Supplementary file 1 — Supplementary Material 1. [file 12944_2025_2694_MOESM1_ESM.pdf]

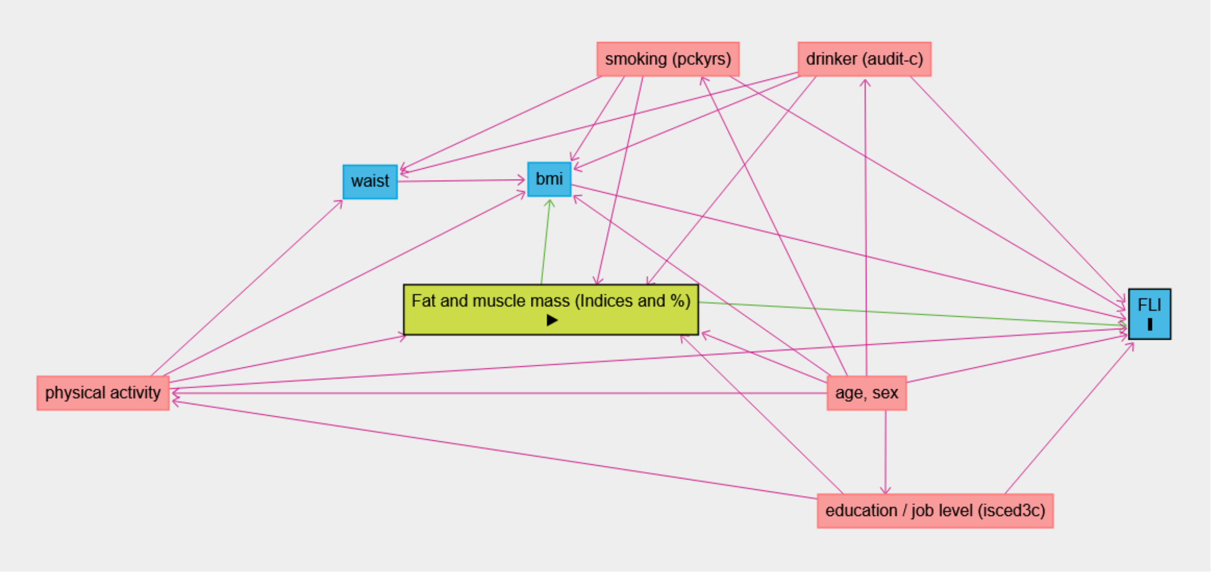

Supplementary Figure S1: A Directed acyclic graph (DAG) for the relationships of fat and skeletal muscle mass indices or percentages with fatty liver index (FLI).

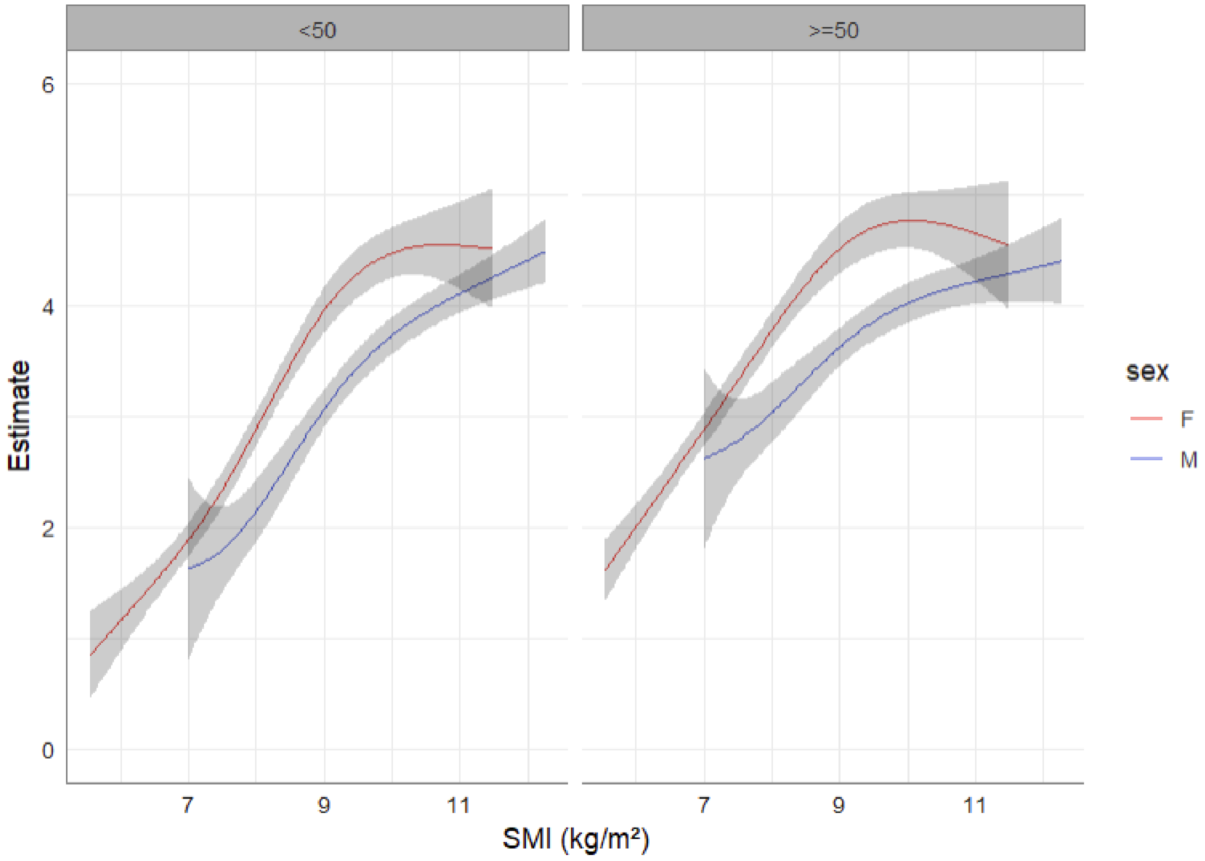

Supplementary Figure S2: Associations between skeletal muscle mass index (SMI, kg/m^2) and fatty liver index (FLI), by gender and age category (below versus above age 50 years)

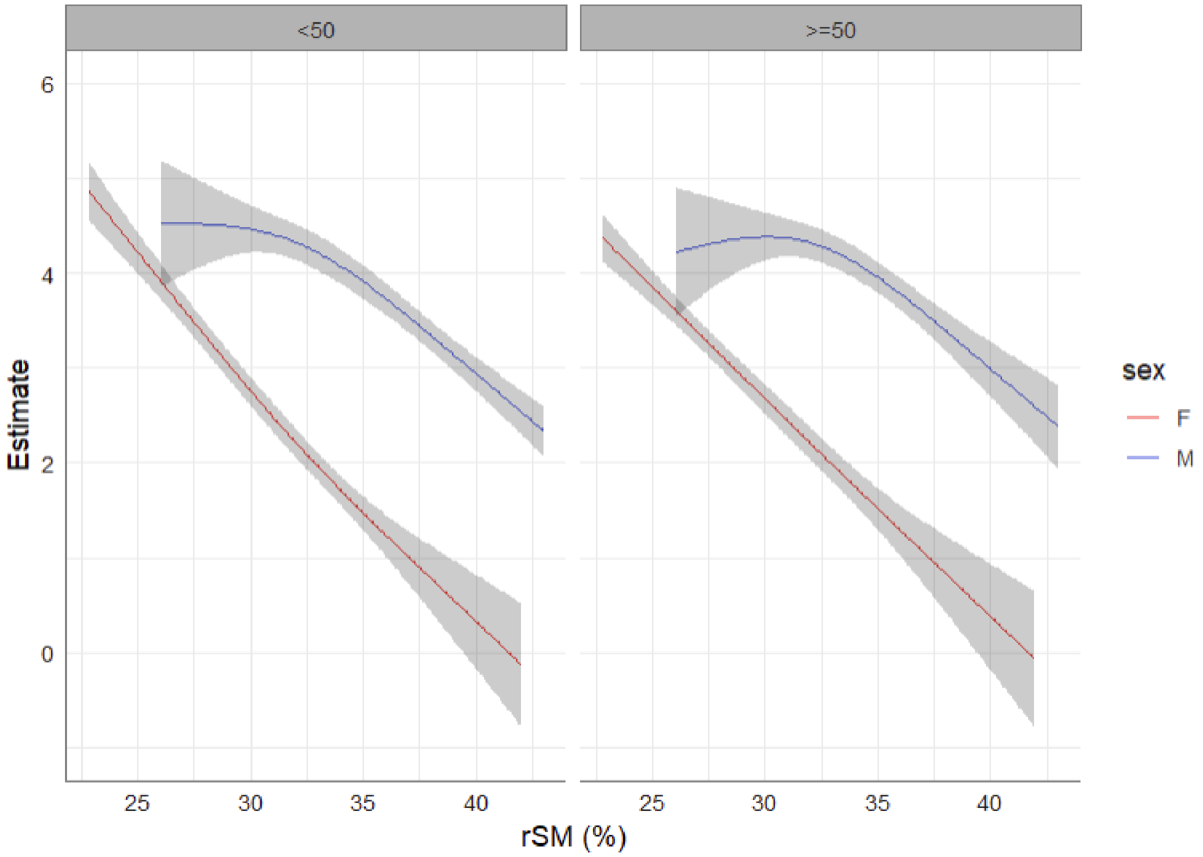

Supplementary Figure S3: Associations between relative skeletal muscle mass (rSM, % b.w.) and fatty liver index (FLI), by gender and age category (below versus above age 50 years)

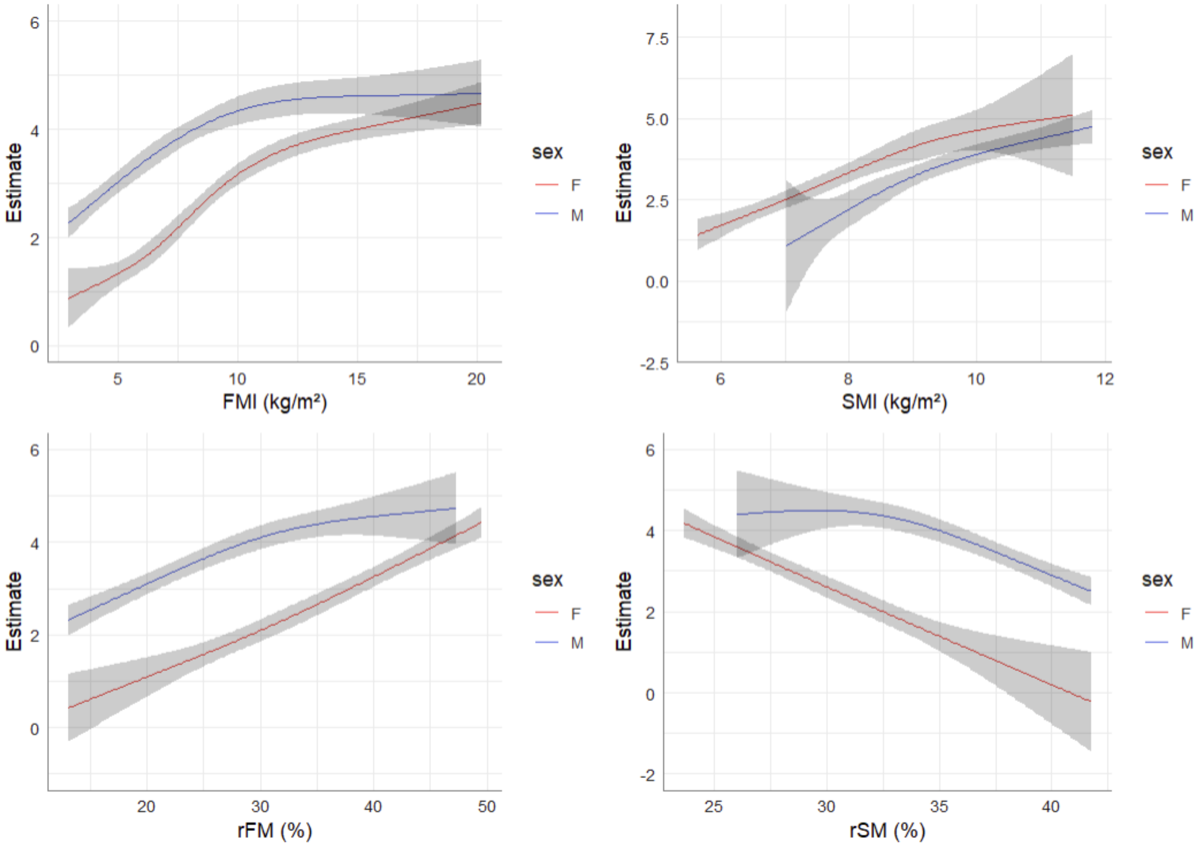

Supplementary Figure S4: Sensitivity analysis: Associations of fat mass index (FMI), skeletal muscle mass index (SMI), relative fat mass (rFM), and skeletal muscle mass (rSM) with fatty liver index (FLI) stratified by gender, using data only from the MEIA study.

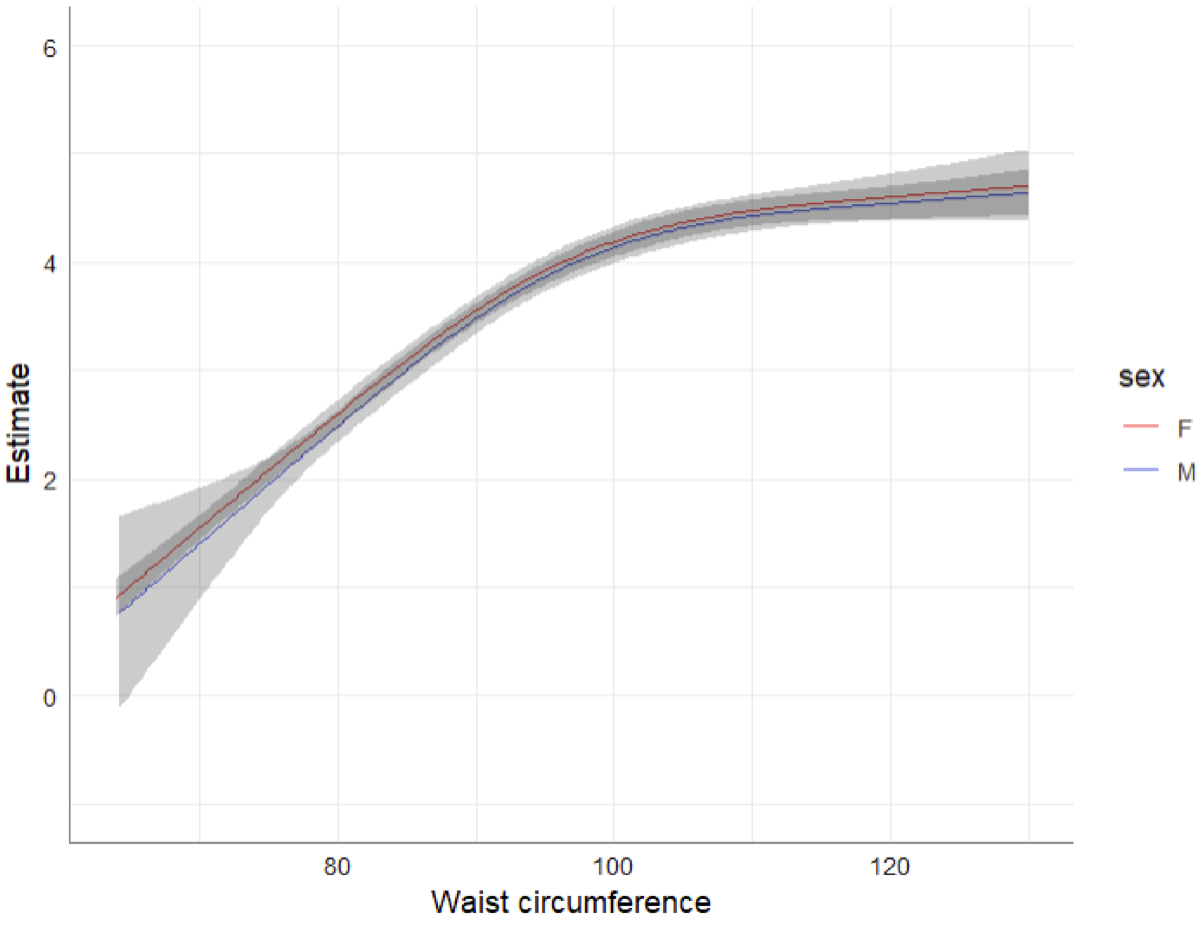

Supplementary Figure S5: Association between waist circumference and fatty liver index (FLI).
