# Supplementary material for: Body muscle mass versus fat mass: gender-specific associations with NAFLD and liver fibrosis
Source: Lipids Health Dis. 2025 Sep 9;24:278. doi: 10.1186/s12944-025-02694-4 (PMC12418690; doi:10.1186/s12944-025-02694-4)
Supplement: Supplementary file 2 — Supplementary Material 2. [file 12944_2025_2694_MOESM2_ESM.pdf]

Supplementary Table S1. Characteristics of the study participants of the MEGA study, stratified by gender.

Continuous variables are given as median and interquartile range and categorical variables are presented as absolute and relative frequencies.

Characteristics	Total	Females	Males	
	n=202	n=143	n=59	p value *
Age (years)	49 (39; 56)	48 (39; 54)	48 (37; 59.5)	0.218
BMI (kg/m²)	24.97 (22.63; 33.16)	24.21 (21.59; 32.13)	30.33 (24.4; 35.3)	<0.001
Waist circumference (cm)	87 (75; 107)	81 (72.25; 99.75)	106 (86.5; 120.5)	<0.001
Fat mass (kg)	24.37 (16.43; 39.61)	23.70 (16.90; 40.77)	29.25 (15.4; 38.5)	0.863
Relative fat mass (%)	34.29 (26.77; 41.04)	35.72 (28.32; 45.12)	29.58 (19.03; 35.21)	<0.001
Fat mass index (FMI)	8.64 (5.83; 13.8)	8.68 (6.03; 14.93)	8.62 (4.41; 11.88)	0.102
Skeletal muscle mass (kg)	23.66 (19.57; 30.18)	21.19 (18.71; 24.30)	34.25 (31.02; 37.05)	<0.001
Relative skeletal muscle mass (%)	30.71 (27.63; 33.73)	29.44 (26.12; 32.15)	34.20 (32.46; 39.49)	<0.001
Skeletal muscle mass index (SMI)	8.19 (7.04; 9.87)	7.40 (6.85; 8.68)	10.31 (9.62; 11.50)	<0.001
Fat mass to muscle mass ratio	1.09 (0.79; 1.46)	1.22 (0.89; 1.74)	0.85 (0.49; 1.08)	<0.001
Fatty liver index (FLI)	19.83 (6.27; 84.69)	12.71 (4.35; 65.37)	75.99 (17.36; 95.09)	<0.001
Liver pressure	5.24 (4.90; 5.78)	5.24 (4.9; 5.7)	5.34 (4.91; 6.12)	0.240
Triglycerides (mg/dl)	82.0 (60; 116.0)	76.5 (57.2; 103.7)	101 (71; 144)	<0.001
γ-Glutamyl transferase (U/l)	18.0 (13; 25.0)	15.0 (12.0; 21.0)	27.0 (21;.47.5)	<0.001
AUDIT-C (No)	3 (2; 4)	2 (2; 3)	3 (2; 5)	<0.001
Smoking (Pack-years)	0 (0; 4.13)	0 (0; 2.5)	0.83 (0; 5.45)	0.012
Smoking				0.051**
Current, (n, %)	24 (11.9)	17 (11.9)	7 (11.9)	
Never, (n, %)	107 (53.0)	83 (5.8)	24 (40.7)	
Previous, (n, %)	71 (35.1)	43 (30.1)	28 (47.5)	
Obesity				0.006**
BMI ≥30 kg/m²) (n, %)	75 (37.1)	44 (30.8)	31 (52.5)	
BMI <30 kg/m²) (n, %)	127 (62.9)	99 (69.2)	28 (47.5)	
Diabetes				
Yes, (n, %)	9 (4.5)	4 (2.8)	5 (8.5)	0.161**
No, (n, %)	193 (95.5)	139 (97.2)	54 (91.5)	

Education				0.511**
High, (n, %)	65 (32.2)	48 (33.6)	17 (28.8)	
Middle, (n, %)	137 (67.8)	95 (66.4)	42 (71.2)	
Low, (n, %)	- (-)	- (-)	- (-)	

AUDIT-C, Alcohol use disorders identification test consumption; BMI, Body mass index; Pack-years, Packs per day x number of years smoked;
* Wilcoxon Rank Sum test, if not otherwise specified; ** Pearson's Chi-squared test

Supplementary Table S2. Characteristics of the study participants of the MEIA study, stratified by gender.

Continuous variables are given as median and interquartile range and categorical variables are presented as absolute and relative frequencies.

Characteristics	Total	Females	Males	
	n=594	n=336	n=258	p value *
Age (years)	49 (34; 60)	49 (34; 58)	50 (34; 61.0)	0.485
BMI (kg/m²)	25.59 (22.64; 29)	24.33 (21.23; 28.3)	26.75 (24.55; 29.45)	<0.001
Waist circumference (cm)	87 (76; 100)	79 (71; 91)	96 (86; 103)	<0.001
Fat mass (kg)	21.95 (16.65; 29.65)	22.37 (16.75; 31.27)	21.64 (16.62; 28.72)	0.260
Relative fat mass (%)	30.21 (24.69; 36.81)	34.35 (28.48; 40.96)	26.22 (21.56; 30.85)	<0.001
Fat mass index (FMI)	7.55 (5.63; 10.27)	8.25 (6.11; 11.20)	6.94 (5.23; 9.02)	<0.001
Skeletal muscle mass (kg)	23.63 (19.58; 30.44)	19.93 (18.02; 21.91)	30.98 (28.04; 33.79)	<0.001
Relative skeletal muscle mass (%)	32.59 (28.64; 35.92)	29.44 (26.72; 32.38)	35.99 (33.71; 38.21)	<0.001
Skeletal muscle mass index (SMI)	8.19 (7.13; 9.58)	7.26 (6.69; 7.99)	9.64 (8.93; 10.40)	<0.001
Fat mass to muscle mass ratio	0.93 (0.69; 1.23)	1.17 (0.87; 1.51)	0.73 (0.56; 0.91)	<0.001
Fatty liver index (FLI)	24.93 (7.64; 62.45)	12.41 (4.87; 37.84)	48.16 (20.32; 73.66)	<0.001
Triglycerides (mg/dl)	89.5 (68; 128.7)	81 (65; 114)	105 (75.25; 145)	<0.001
γ-Glutamyl transferase (U/l)	19.0 (14; 29.0)	15.0 (12.0; 22.0)	25.0 (19.0; 37.0)	<0.001
AUDIT-C (No)	3 (1; 4)	2 (1; 4)	4 (2; 5)	<0.001
Smoking (Pack-years)	0 (0; 7.9)	0 (0; 6.0)	0.05 (0; 10.75)	0.096
Smoking				0.463**
Current, (n, %)	90 (15.8)	48 (14.6)	42 (17.4)	
Never, (n, %)	300 (52.6)	180 (54.7)	120 (49.8)	
Previous, (n, %)	180 (31.6)	101 (30.7)	79 (32.8)	
Obesity				0.272**
BMI ≥30 kg/m²) (n, %)	129 (21.7)	67 (19.9)	62 (24.0)	
BMI <30 kg/m²) (n, %)	465 (78.3)	269 (80.1)	196 (76.0)	
Diabetes				0.385**
Yes, (n, %)	8 (1.3)	3 (0.9)	5 (1.9)	
No, (n, %)	207 (34.8)	123 (36.6)	84 (32.6)	
Education				0.199**

High, (n, %)	233 (39.2)	123 (36.6)	110 (42.6)
Middle, (n, %)	333 (56.1)	199 (59.2)	134 (51.9)
Low, (n, %)	28 (4.7)	14 (4.2)	14 (5.4)

AUDIT-C, Alcohol use disorders identification test consumption; BMI, Body mass index; Pack-years, Packs per day x number of years smoked;
* Wilcoxon Rank Sum test, if not otherwise specified; ** Pearson's Chi-squared test

Supplementary Table S3: Sensitivity analysis: Estimates and 95% confidence intervals from multivariable gamma regression models for the relationships between body composition variables (FMI, SMI, rFM, rSM, FMR) with fatty liver index (FLI) in participants of the **MEIA study only**. The estimates on the exponential scale can be interpreted as percentage change with the 1 as reference. P values for the estimated effects were Bonferroni adjusted. (Results of the non-linear associations are graphically shown in Figures 1-3)

Exposure	Outcome	Estimate (95% CI)	P	P gender-interaction	P age-interaction
FMI	FLI	non-linear (4 knots)	<0.0001	<0.0001	0.3679
rFM	FLI	non-linear (4 knots)	<0.0001	0.0044	0.3115
SMI	FLI	non-linear (3 knots)	<0.0001	0.8015	0.0247
rSM	FLI	non-linear (3 knots)	<0.0001	0.0138	0.0050
FMR	FLI	non-linear (3 knots)	<0.0001	0.0110	0.1821

Supplementary Table S4: Estimates and 95% confidence intervals from multivariable gamma regression models for the relationship between waist circumference (WC) with fatty liver index (FLI) and liver fibrosis (liver pressure).

The estimates on the exponential scale can be interpreted as percentage change with the 1 as reference.

Exposure	Outcome	Estimate (95% CI)	P	P gender-interaction	P age-interaction
WC	FLI	non-linear (4 knots)	<0.0001	0.9507	0.1387
WC	Liver Pressure	1.000 (0.994; 1.006)	0.8658	0.3266	0.4287
